# Using metabolic networks to predict cross-feeding and competition interactions between microorganisms

**DOI:** 10.1128/spectrum.02287-23

**Published:** 2024-03-20

**Authors:** Claudia Silva-Andrade, María Rodriguez-Fernández, Daniel Garrido, Alberto J. M. Martin

**Affiliations:** 1 Programa de Doctorado en Genómica Integrativa, Vicerrectoría de Investigación, Universidad Mayor, Santiago, Chile; 2 Laboratorio de Redes Biológicas, Centro Científico y Tecnológico de Excelencia Ciencia & Vida, Fundación Ciencia & Vida, Santiago, Chile; 3 Institute for Biological and Medical Engineering, Schools of Engineering, Medicine and Biological Sciences, Pontificia Universidad Católica de Chile, Santiago, Chile; 4 Department of Chemical and Bioprocess Engineering, School of Engineering, Pontificia Universidad Católica de Chile, Santiago, Chile; 5 Escuela de Ingeniería, Facultad de Ingeniería, Arquitectura y Diseño, Universidad San Sebastián, Santiago, Chile; University of Michigan-Ann Arbor, Ann Arbor, Michigan, USA

**Keywords:** bacterial interaction, cross-feeding, competition, machine learning

## Abstract

Understanding the interactions between microorganisms and their impact on bacterial behavior at the community level is a key research topic in microbiology. Different methods, relying on experimental or mathematical approaches based on the diverse properties of bacteria, are currently employed to study these interactions. Recently, the use of metabolic networks to understand the interactions between bacterial pairs has increased, highlighting the relevance of this approach in characterizing bacteria. In this study, we leverage the representation of bacteria through their metabolic networks to build a predictive model aimed at reducing the number of experimental assays required for designing bacterial consortia with specific behaviors. Our novel method for predicting cross-feeding or competition interactions between pairs of microorganisms utilizes metabolic network features. Machine learning classifiers are employed to determine the type of interaction from automatically reconstructed metabolic networks. Several algorithms were assessed and selected based on comprehensive testing and careful separation of manually compiled data sets obtained from literature sources. We used different classification algorithms, including K Nearest Neighbors, XGBoost, Support Vector Machine, and Random Forest, tested different parameter values, and implemented several data curation approaches to reduce the biological bias associated with our data set, ultimately achieving an accuracy of over 0.9. Our method holds substantial potential to advance the understanding of community behavior and contribute to the development of more effective approaches for consortia design.

**IMPORTANCE:**

Understanding bacterial interactions at the community level is critical for microbiology, and leveraging metabolic networks presents an efficient and effective approach. The introduction of this novel method for predicting interactions through machine learning classifiers has the potential to advance the field by reducing the number of experimental assays required and contributing to the development of more effective bacterial consortia.

## INTRODUCTION

A microbial consortium is a group of different species or strains of microorganisms that interact to execute specific behaviors. Within microbial consortia, different types of interactions occur among microorganisms and their neighbors (1). In this way, cross-feeding and competition interactions allow for the optimization of parallel metabolic processes of different microorganisms to increase productivity, efficiently consume certain nutrients, or maintain consortium stability against environmental perturbations over time(2–4). Cross-feeding interactions may be unidirectional, where one microorganism benefits and grows by utilizing secreted metabolites from another, or bidirectional, involving a reciprocal exchange of metabolites among different microorganisms in the community(5–9⁠). Notably, understanding these interactions is key for understanding individual microorganism behavior within a consortium and, even more importantly, for unraveling the collective behavior of an entire community of microorganisms.

Microbial consortia engineering (MCE) has recently evolved into an established scientific discipline on its own (10⁠). The principal objective in this field is to create communities that exhibit stability over time, enhanced productivity of specific metabolites, and improved metabolic functionalities (1, 2, 11–13). In essence, MCE seeks to design consortia of microorganisms with specific properties of interest. The most commonly used MCE methodology involves assembling the metabolism of different microorganisms to achieve a particular behavior that promotes interactions among the different cells and their environment (12, 13). Other approaches, such as ecology-based models (14) and ODE-based (15–17) mechanistic models, do not rely on the whole genome. It is widely acknowledged that knowledge about each isolated microorganism is insufficient to explain the behavior and properties of consortia (3, 4, 18–21⁠). Therefore, understanding the behavior of a microbial consortium is contingent on determining the relationships between the microorganisms within it and how these relationships influence the community’s behavior (13, 22–24). Moreover, when aiming to design a consortium with a specific behavior, it is necessary to previously identify the key microbial species that contribute to active beneficial processes and the positive interactions supporting the growth and stability of these populations in the community (25⁠).

Regardless of the method used for MCE, genomic information is deemed essential for achieving optimal results. Advances in genome sequencing technologies have enabled the comprehensive description of the metabolism of different organisms at a whole-genome scale (26, 27⁠). Over the last 20 years, metabolic network reconstruction has significantly expanded its range of applications (20, 28). For instance, current uses of metabolic networks include understanding various metabolic processes in a microorganism to optimize the production of a particular metabolite (29–32) or to comprehend the metabolic properties of different microorganisms (22, 28, 31⁠).

The reconstruction of a metabolic network involves using an organism’s genomic information to understand its metabolism. This process typically comprises several consecutive steps (24⁠). In the first step, a draft reconstruction of the network is generated based on the genome annotation to identify a collection of metabolic functions encoded in the genome. Several tools can be employed to create the initial reconstruction of a metabolic network (33⁠). The second step involves the manual curation of the draft network, requiring a meticulous review of each enzyme and reaction in the metabolic network based on previous knowledge of the organism and known metabolic reactions. In the third step of reconstruction, the metabolic network is converted into mathematical computable functions or models, usually written in a standardized format. The last step involves the verification, evaluation, and validation of the metabolic model to identify missing metabolic functions, often necessitating a repetition of the second and third steps (23⁠). Notably, there is also an automated reconstruction tool for genome-scale metabolic models, CarveMe, which employs an innovative top-down reconstruction approach (34)⁠.

Although genome-scale metabolic reconstruction has different applications, the majority of published uses are related to improving the understanding of a bacteria’s metabolism at a molecular level and increasing the production of certain metabolites in a single organism (25, 29).

Different strategies to define, understand, and characterize cross-feeding interactions between bacteria are based on experimental or mathematical approaches (34⁠). For instance, to predict cross-feeding interaction in the gut microbiome, GutCP (14)⁠ combines machine learning techniques with an ecological-guided model of the microbiome. At the same time, MICOM (35)⁠ integrates taxonomic abundance based on metagenomic samples with dietary constraints to generate personalized metabolic models. Another tool for analyzing metabolic interactions and microbial consortia, the Microbiome Modeling Toolbox (36⁠), employs metagenomic data and microbial metabolic reconstructions as input. Baldini et al. (37⁠) used nutrient dynamics with a coarse-grained description of cell metabolism, integrating an ecology model for the population to develop cross-feeding models. Freilich et al. (7) developed a novel methodology to predict overall potential interspecies interactions in different environments using metabolic reconstruction and ecological co-occurrence patterns. In their study, the authors simulated the possible competitive and cooperative interactions between pairs of bacteria under different growth conditions. It is also relevant to mention tools that predict non-gut microbiome consortia, such as SMETANA (38)⁠, which can predict higher-order community interactions based on *in vitro* media and conditions.

Importantly, these approaches rely on highly curated metabolic networks, often requiring manual intervention, and also demand access to extensive computational infrastructures (35)^⁠^. In this work, we focus on identifying novel cross-feeding and competition interactions between bacteria by developing a new computational approach. This innovative tool, based on a relatively simple machine learning algorithm, employs metabolic networks automatically reconstructed from genome annotations to predict bacterial interactions. Importantly, our method demands lighter computational resources, and once trained, it is usable on standard computers. Initially, we compiled a data set of pairs of bacteria for which their interaction had been previously reported. Subsequently, we explored various methods to automatically reconstruct metabolic networks and encode this information into a linear vector describing pairs of bacteria. Through comprehensive testing, we demonstrate how our relatively simple approach effectively distinguishes between cross-feeding and competition interactions.

## RESULTS

### Data set of predictor

**Fig 1:**
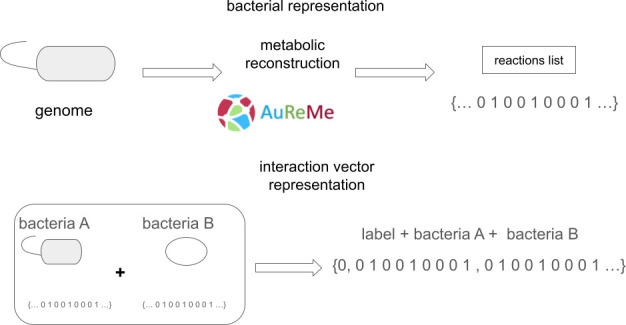
Vector creations. Graphical representation of the creation of data set.

**Fig 2:**
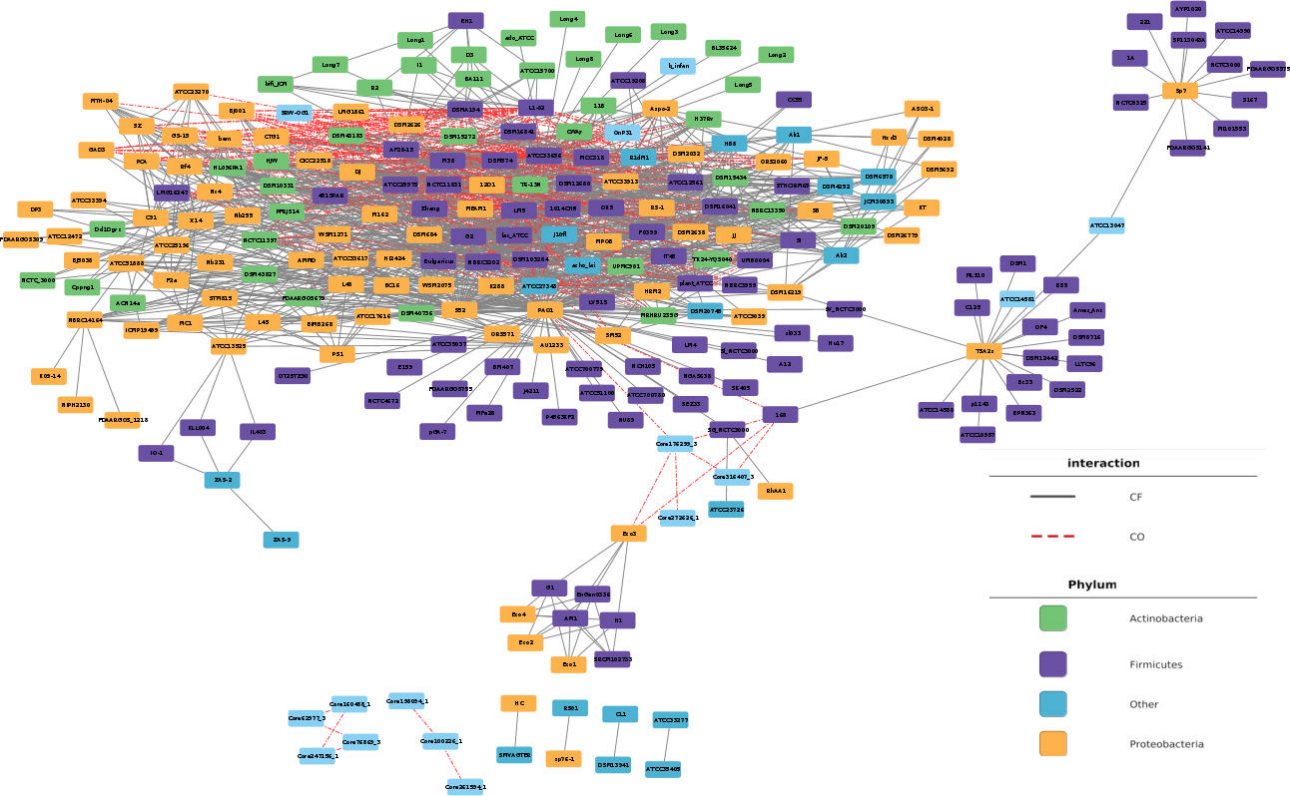
Network representation of the data set employed in this work. As indicated by the color codes, there are 260 microorganisms in this network, 6 archaea and 254 bacteria of different phylum. These microorganisms are connected by 1,326 interactions: 1,053 pairs of cross-feeding and 273 of competition.

We created a data set of cross-feeding and competition interactions using the experimentally validated interactions outlined by Liao et al. (39)⁠ and the NJS16 database (38)⁠ (Fig. 1). This effort resulted in the identification of 1,053 cross-feeders and 273 competitor pairs of bacteria, involving 254 bacteria and 6 archaea with sequenced genomes. Within the bacteria category, 92 belong to the Proteobacteria phylum, 99 to the Firmicutes phylum, 43 to the Actinobacteria phylum, 3 to the Verrucomicrobia phylum, 2 to the Deinococcus-Thermus phylum, 2 to the Tenericutes phylum, 3 to the Chloroflexi phylum, 4 to the Spirochaetes phylum, 3 to the Bacteroidetes phylum, 2 to Cyanobacteria, and 1 bacterial genome belongs to the Fusobacteria phylum (Table S1). These microorganisms and their interactions can be visualized as a network in Fig. 2. We employed AuReme (40)⁠ with default parameters to automatically reconstruct the metabolic network of each genome used in this analysis. Subsequently, we assessed the presence of each reaction in all reconstructed metabolic networks to build feature vectors for training different classification algorithms. On average, there are 793 reactions in the bacterial genome-wide metabolic networks and a total pool of 3,141 different reactions.

**Fig 3:**
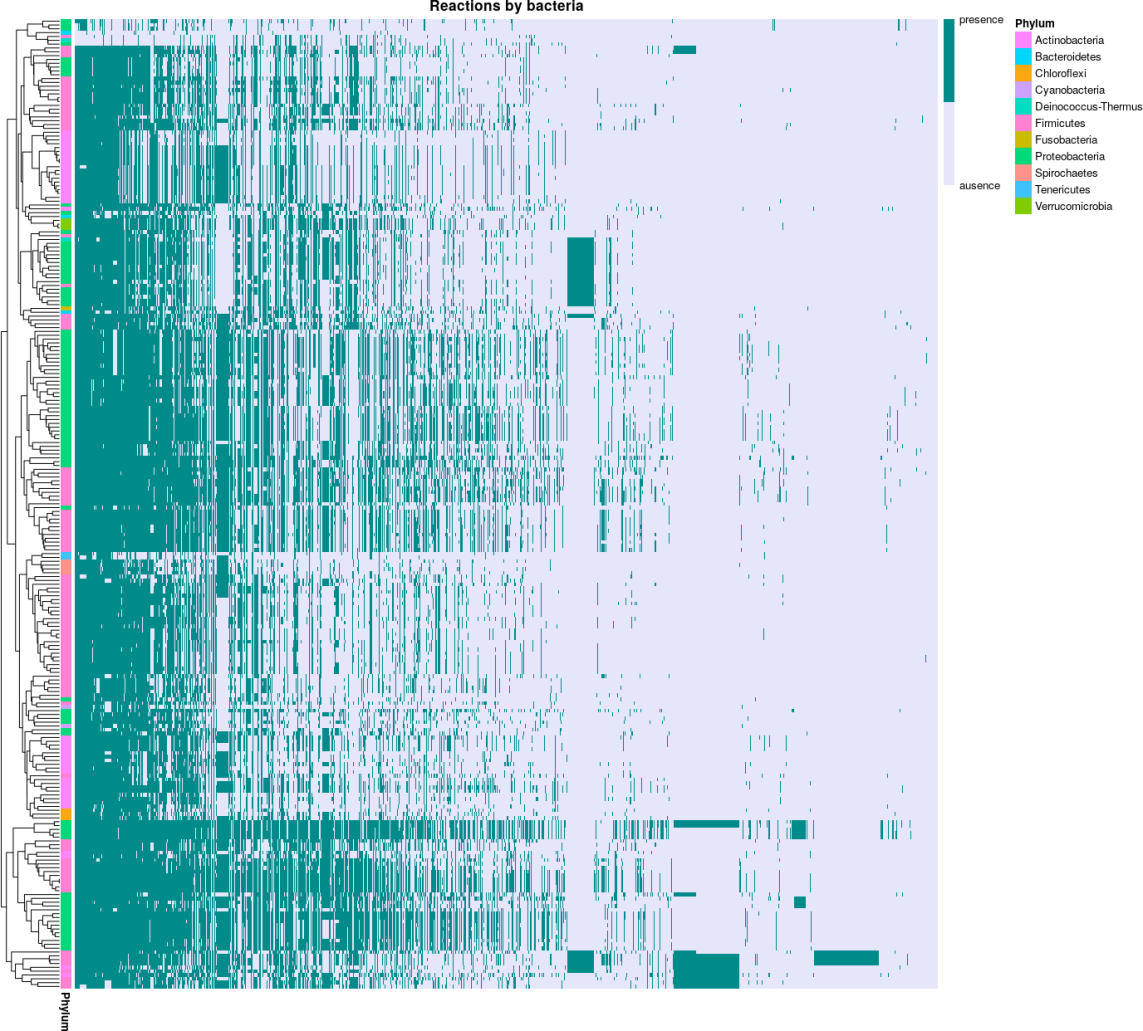
Reactions in bacterial genomes. Presence (blue) or absence (lavender) of reactions in bacterial genomes. The *x*-axis represents different reactions, and the *y*-axis represents the bacterial genomes

The presence or absence of each reaction across all bacterial genomes can be observed in Fig. 3, where the color pattern reflects the distinct metabolism of each bacterium compared to others, showcasing differences in the automatically reconstructed metabolic networks.

Pairs of interacting bacteria are represented by binary vectors where each element signifies the presence or absence of a reaction in each of the two bacteria. Given that these vectors have 2 × 3,141 elements, we included each pair of bacteria that cross-feed each other twice to mitigate the potential bias caused by the position of each bacterium in the reactions. This approach also serves as data augmentation, doubling the number of examples, resulting in 2,106 cross-feeding interactions and 546 competing pairs. Before applying any prediction algorithm, we clustered the 2,652 vectors to minimize the overlap between folds in a cross-validation (41⁠). We employed the K-means algorithm implemented in the Scikit-learn library (42⁠) starting with 10 target clusters until we could create four folds with roughly the same number of pairs of interacting microorganisms. These folds are described in Table 1.

**TABLE 1:** Fold description^*a*^

Fold	Number of examples	Number of competition	Number of cross-feeding
F0	693	123	570
F1	659	148	511
F2	688	175	513
F3	612	100	512

^*a*^ Folds created after clustering the total number of interacting microorganisms to minimize the overlap between each group of examples.

### Identification of cross-feeding interactions using a KNN algorithm

The first algorithm we employed to predict the type of interaction between any pair of bacteria is the KNN algorithm. We used this algorithm as a baseline because it is the simplest supervised classifier. To determine the optimal number of neighbors, *k*, we used 10% of randomly selected data. This subset served as a representative sample of the data, and its usage was chosen for its expediency compared to using the complete data set.

**Fig 4:**
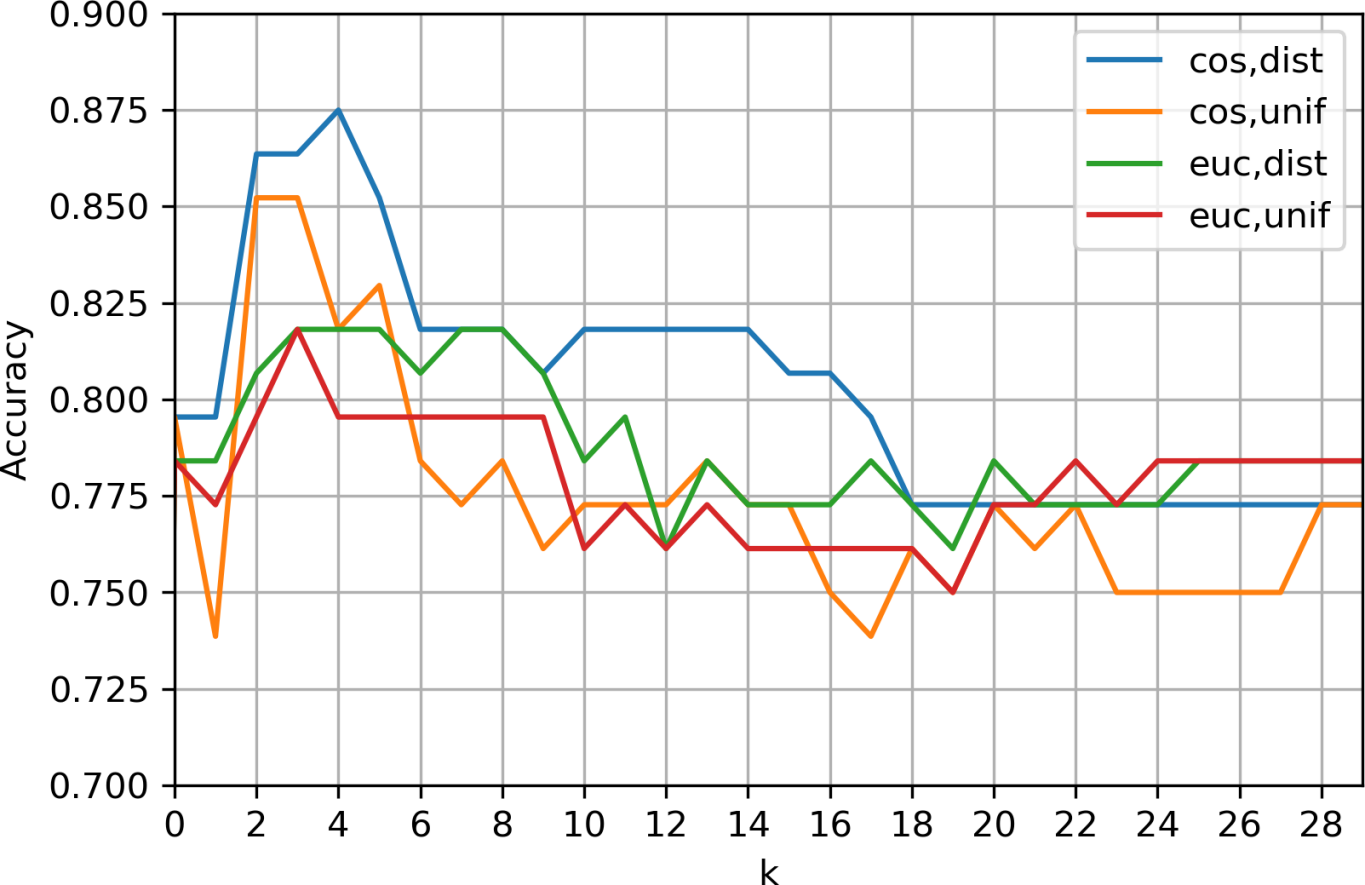
Accuracy by *k* value. Accuracy of the KNN algorithm by *k* value in 10% of total data using 67% as the training set and 33% of the samples in the test set. The *x*-axis represents different *k* values, and the *y*-axis represents the accuracy or percentage of correctly classified samples.

We tested values from 1 to 30, employing 67% of the data for the training set and 33% for the test set. Additionally, we tested both cosine and Euclidean distances considering weighted and non-weighted options. As shown in Fig. 4, we selected a *k* of 3 as it yielded the best results based on the accuracy metric (percentage of correctly classified samples). For the target class “competition,” we obtained a precision of 0.76 and a recall of 0.73. In the case of the target class “cross-feeding,” we attained a precision of 0.95 and a recall of 0.94. It is important to note that a random prediction for the competition class should yield a precision of 0.21, while for cross-feeding, a random prediction would result in a precision of 0.79.

### Machine learning method test

We proceeded to compare the KNN classifier by evaluating its performance against other algorithms for predicting bacterial interactions. Using Scikit-learn, we assessed the performance of the following algorithms: Random Forest Classifier (RF), SVMs (linear, RBF, sigmoid, and polynomial kernels), and XGBoost. Performance metrics, including accuracy, balanced accuracy, precision, recall, and F1 score, were reported in the mean of all folds, as shown in Table 2. Given the unbalanced data set, with approximately four times more cross-feeding samples than competition ones, we opted to report both classes as targets. This approach ensures that any bias arising from the data set’s composition is duly considered. We obtained accuracies between 0.79 and 0.93, indicating that overall, all classifiers are able to differentiate both classes. Nonetheless, upon closer examination of precision, recall, and F1 score, notable differences emerge when each of the two classes is targeted, with lower values observed for competition compared to cross-feeding. Importantly, these differences are less pronounced for XGBoost, which exhibits the best performance across all calculated metrics. It is worth noting that these metrics were obtained through internal cross-validation testing, which tends to be optimistic compared to validation with an external data set.

**TABLE 2:** Comparison of classifier algorithms

	TP_co	FN_co	FN_cf	TP_cf	P_co	R_co	F1_co	P_cf	R_cf	F1_cf	Acc	B acc
KNN *k* = 3	401	145	130	1,976	0.76	0.73	0.74	0.93	0.94	0.93	0.9	0.84
SVM linear	369	177	104	2,002	0.78	0.68	0.72	0.92	0.95	0.93	0.89	0.81
SVM RBF	343	206	163	1,943	0.68	0.63	0.65	0.91	0.92	0.91	0.86	0.78
SVM polynomial	376	170	140	1,966	0.73	0.69	0.71	0.92	0.93	0.88	0.88	0.81
SVM sigmoid	158	388	176	1,930	0.47	0.29	0.36	0.83	0.92	0.87	0.79	0.6
XGBoost	**445**	**101**	**80**	**2,026**	**0.85**	**0.82**	**0.83**	**0.95**	**0.96**	**0.96**	**0.93**	**0.9**
RF	429	117	119	1,987	0.78	0.79	0.78	0.94	0.94	0.94	0.91	0.86

^*a*^ This table reports common classification metrics calculated using both cross-feeding (cf) and competition (co) as target class and by performing a fourfold cross-validation on the folds made by clustering samples. TP_co are correctly classified competition examples, TP_cf are correctly classified cross-feeding pairs, and FN_co and FN_cf are competition examples classified as cross-feeding (false negatives co) and cross-feeding examples misclassified as competition, respectively. Precision (P), recall (R), and F1 score metrics are reported for the two classes, while accuracy (Acc) considers both equally. Bold indicates the best result in our comparison. Finally, B acc is the balanced accuracy.

**Fig 5:**
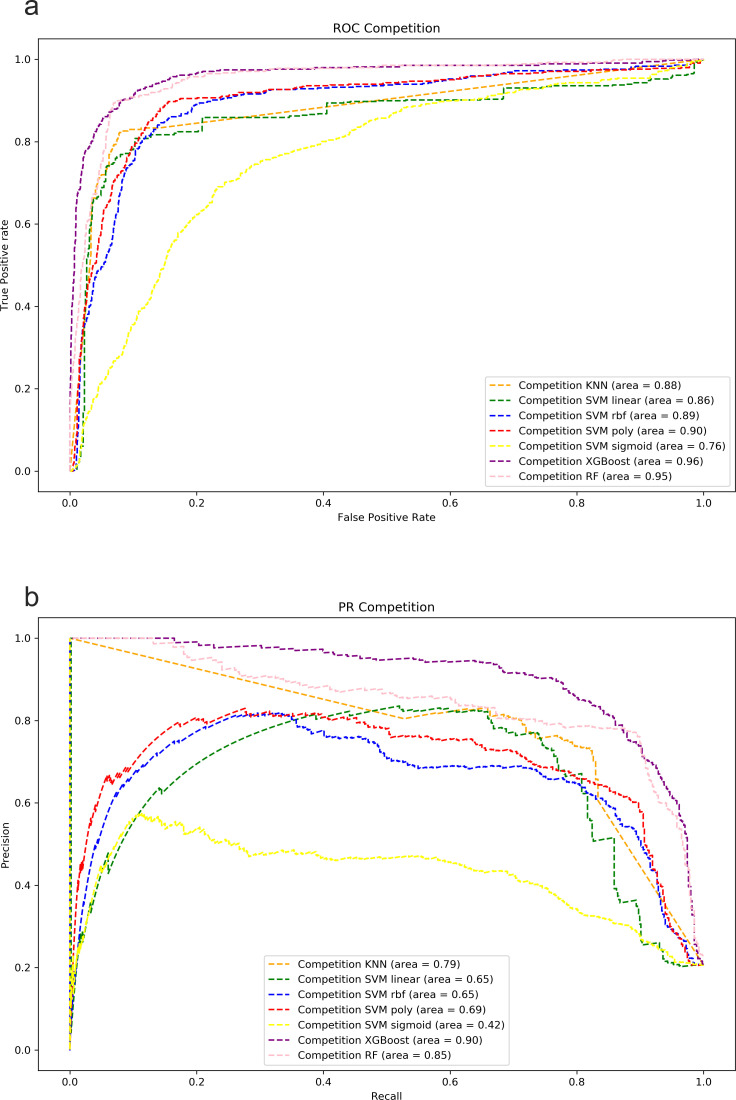
Area under the ROC and PR curves using as target class competition examples. (**a**) ROC curves for the competition class and (b) PR curves for the competition class.

To further establish the ranking between these classifiers, we constructed ROC and PR curves and calculated areas under these curves. These results are shown in Table 3; Fig. 5. Similar to the classification metrics, the XGBoost algorithm exhibited the best areas under the curves. As expected, we also observed differences in the PR areas when using cross-feeding or competition classes as targets, with greater values for the cross-feeding class. Examining the curves with the competition class as the target (refer Fig. 5), it is evident that the XGBoost algorithm outperforms all the other methods tested.

**TABLE 3:** Area under ROC and PR curves^*a*^

	ROC AUC_co	ROC AUC_cf	PR AUC_co	PR AUC_cf
KNN	0.88	0.88	0.79	0.97
SVM linear	0.86	0.86	0.65	0.92
SVM RBF	0.89	0.89	0.65	0.95
SVM polynomial	0.9	0.9	0.69	0.95
SVM sigmoid	0.76	0.76	0.42	0.91
XGBoost	**0.96**	**0.96**	**0.9**	**0.98**
RF	0.95	0.95	0.85	**0.98**

^*a*^ Areas for each of the compared algorithms using as target the two classes, cross-feeding (cf) and competition (co). Bold indicates the best results.

### Test on Freilich et al. data set

We used our predictor on the bacterial data set, composed of 117 different strains described by Freilich et al. (7⁠) (one of 118 strains of the study is not available in the public database) to predict the interactions in pairs of bacteria. Our predictor identified 1,035 competition and 5,751 cross-feeding interactions between bacterial pairs.

The Freilich et al. data set included 186 competition interactions and 2,401 cross-feeding interactions. They reported 4,370 pairs of bacteria without possible interactions. Considering the interaction data from the Freilich data set (discarding NI), we obtained an accuracy of 0.72, F1 score of 0.85, and recall of 0.83.

**Fig 6:**
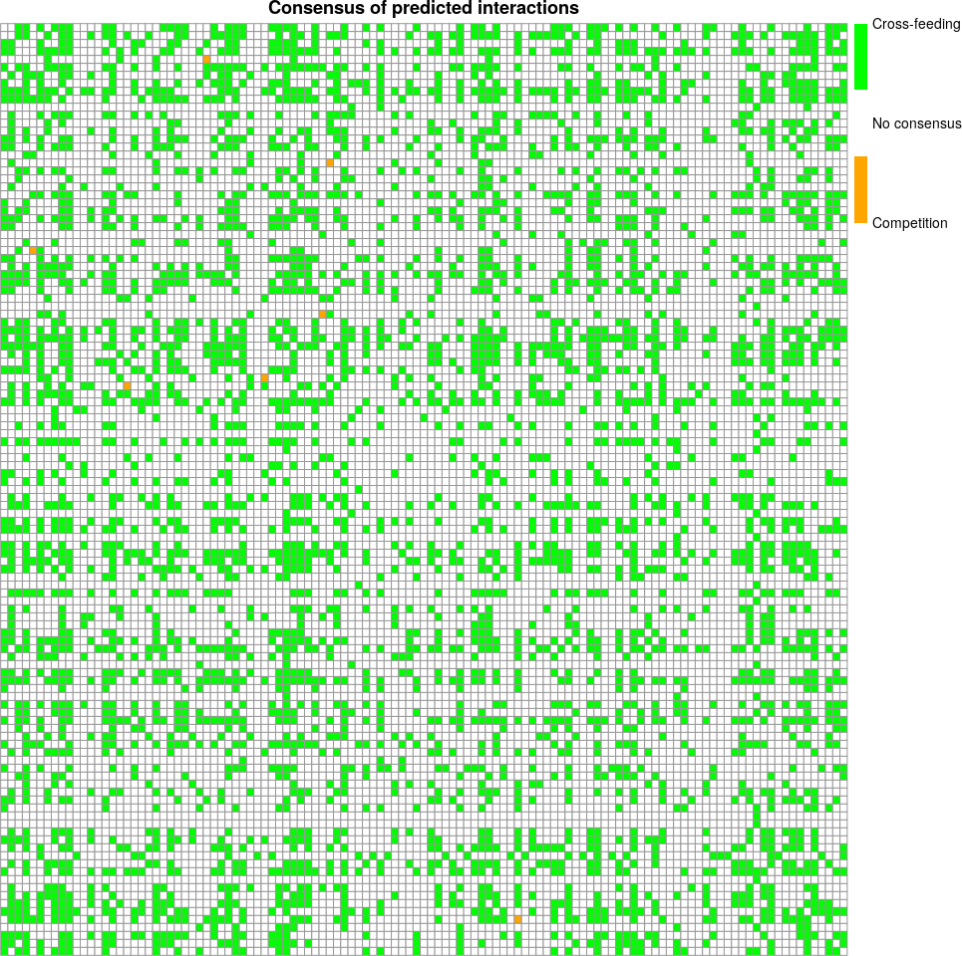
Consensus in predicted interactions. Cross-feeding (green), competition (orange), and no consensus (white) interaction between pairs of bacterial genomes between transformed potential cooperation score (PCPS) matrix from Freilich et al. study (7⁠) and results of our predictor. The *x*-axis and the *y*-axis represent the bacterial strain evaluated.

We compared our results with those obtained by Freilich et al. (7⁠) and obtained a consensus of three competitions and 1,892 cross-feeding interactions between the methods. In the matrix (Fig. 6), each position represents a comparison between the results of our predictor and those of Freilich et al. (7⁠), represented in orange when both results were competitive interaction, green when the result was cross-feeding interaction, and white when there was no consensus interaction between the methods.

## DISCUSSION

In this work, we developed a novel bacterial interaction predictor based on metabolic network features using different classifier algorithms. The utilization of metabolic information for analyzing and assembling microbial consortia has been considered in different studies (7, 28, 43–47). Motivated by this, we aimed to create a model capable of predicting the type of interaction between pairs of microorganisms with minimal information and resources while still effectively describing a bacterium.

We curated a data set of cross-feeding and competition interactions sourced from the literature (48, 49). This data set includes a pool of 3,141 reactions obtained from automatically generated metabolic networks for 244 bacteria and 6 archaea. On average, each genome exhibits 793 reactions, showcasing a distinct pattern for each microorganism, reinforcing the differences among the analyzed microorganisms. The data set contains 1,053 cross-feeding and 273 competing pairs of microorganisms. To mitigate biases stemming from the order of microorganisms in the numeric vectors employed for training classification algorithms, we implemented data augmentation. This process involved creating new vectors for each unique combination of bacteria A and B, considering both AB and BA arrangements.

The data set forms the foundation of our method and was used to train several machine learning classification algorithms. Given the relatively small number of examples in our data set, we opted to perform a fourfold cross-validation instead of dividing it into test and training sets. To minimize similarities between folds, we applied k-means clustering. This approach ensures that the reported performance is based on training and testing data sets that are as dissimilar as possible, decreasing the chances of overfitting.

We initiated our exploration with a KNN algorithm, using the optimal k as determined by the accuracy of 10% of randomly selected data, using 67% for training and 33% for testing. Notably, we achieved promising results with a precision of 0.76 and recall of 0.73 when competition was the target class, considering that a random prediction should give a precision of 0.21. For cross-feeding as the target class, we obtained a precision of 0.95 and a recall of 0.94, surpassing the expected precision of 0.79 for a random prediction. This result obtained with the simplest classification algorithm is very promising, indicating the ability to correctly define the interactions between two microorganisms based on automatically annotated metabolic reactions from their genome.

To discard potential artifacts from the machine learning algorithm and explore more complex classification method, we compared the baseline KNN with the algorithms: RF, SVMs (using linear, polynomial, RBF, and sigmoid kernels), and XGBoost. For each algorithm, we consistently observed worse results using competition as the target class compared to cross-feeding (the most numerous class). This difference suggests that competition interactions in our data set might not be sufficiently representative to distinguish between cross-feeding and competition interactions. Another possibility is that competition interactions are more difficult to identify, but we would require larger data sets to validate this hypothesis. Nonetheless, the standout performer across all assessments was XGBoost, which confirms its efficacy in handling unbalanced data sets.

There are other methods available to predict bacterial interactions. One method that uses machine learning to predict cross-feeding interactions in the gut microbiome is GutCP (14)⁠. This method combines an ecological-guided model of the microbiome with machine learning techniques. Another approach, MICOM (36)⁠⁠, integrates taxonomic abundance based on metagenomic samples with dietary constraints to generate personalized metabolic models. Furthermore, the Microbiome Modeling Toolbox (37)⁠ uses metagenomic data and microbial metabolic reconstructions to analyze metabolic interactions and microbial consortia.

In contrast to these methods, our approach relies solely on metabolic network information automatically generated from the annotated genome of two microorganisms, making it the most straightforward and cost-effective approach available. The study of Freilich et al. (7⁠) also used metabolic reconstruction along with ecological co-occurrence patterns to predict and simulate interactions between pairs of bacteria under different conditions. For this reason, we used our predictor to forecast the interactions and compared the PCPS (potential cooperation scores) matrix (with interactions predicted) with the species combinations created by them. We obtained 1,895 pairs of consensus interactions with their study, constituting approximately one-third of the complete data set. Although the consensus corresponds to only one-third of the complete data set, it should be noted that our method enhances the accuracy and quality of the information used, as we have used real interactions between pairs of bacteria rather than simulations, and this information has increased in recent years. Additionally, if we only considered the interaction data from the Freilich et al. data set (discarding NI), we obtained an accuracy of 0.72, an F1 score of 0.85, and a recall of 0.83. Since 2017, the NJS16 database has housed a collection of experimentally validated interactions between pairs of bacteria. The availability of experimentally validated interaction data presents immense potential for understanding the interactions between pairs of bacteria in a community. However, unraveling the behavior of microbial consortia remains a challenge that computational approaches hold promise in addressing in the future. Additionally, another important limitation of our method is that the predictions do not offer insights into the specific metabolites exchanged between pairs or resources over which different genotypes compete in case of competition or shared in the context of the cross-feeding case.

We must stress that our approach relies solely on literature data that has undergone experimental validation. Therefore, there is a potential bias in the representativeness of the strains used in the examples. Nevertheless, our trained models are compatible with standard computers and can predict interactions within minutes.

Our method, grounded in experimentally validated interactions between pairs of bacteria rather than simulations, has yielded promising results, suggesting the encouragement of our machine learning approach. However, there is room for improvement and refinement, particularly as more validated information, especially concerning competition examples, becomes available.

In conclusion, unraveling the interactions among microorganisms within a consortium and understanding their impact on community behavior are paramount. The integration of metabolic networks and machine learning has facilitated rapid data analysis, enabling the evaluation of new methodologies to expand our knowledge of microbial community relationships.

The inclusion of information regarding the culture medium in our method for predicting bacterial interactions could, in the future, enhance the predictive ability by capturing the influence of the environment on metabolic responses. By considering different environmental conditions, our model could simulate more realistic scenarios and adapt to changes in the environment, thereby improving predictive accuracy. This addition allows us to explore indirect interactions and provide a more complete representation of complex microbial dynamics, which is especially valuable for practical applications in fields such as microbiological research, biotechnology, and health.

The concept of representing metabolic networks as inputs for machine learning has been previously explored by DiMucci et al. (50⁠). They employ a representation of bacterial interactions, incorporating features derived from relevant traits, such as metabolic functions, presence/absence of specific genes, or any other pertinent trait by each bacterium, to train machine learning algorithms.

In this study, we introduced a novel machine learning method that utilizes automatically reconstructed metabolic networks to predict cross-feeding and competition interactions between pairs of microorganisms. While there is still room for improvement, our method exhibits excellent performance in distinguishing between cross-feeding and competitive interactions. This tool holds the potential to aid the selection of microbial consortia components and advance our understanding of microbiota relationships.

## MATERIALS AND METHODS

### Bacterial genome for predictor

Our analysis included 204 bacterial genomes and 6 genomes from archaea, gathered from a literature review (48, 49), with most of them extracted from the NJS16 database (49⁠), and an additional 61 bacterial genomes sequenced in our laboratory (see Table S1). Among these microorganisms, a total of 1,326 interactions were reported, comprising 1,053 pairs involved in cross-feeding and 273 pairs in competition interactions.

### Generation of genome-based metabolic models

AuReMe (40)⁠ was used to reconstruct the metabolic network for each organism in this project. The orthology-based reconstruction method, which uses OrthoFinder (51)⁠ software within the Aureme docker, was applied. Metabolic maps from AGORA (19) served as a reference, with the selection of the representative microorganism being based on its closest phylogenetic relationship.

### Vector representation of the metabolic network and interacting microorganisms

In the metabolic maps obtained from AGORA (19⁠), a total of 5,395 different reactions were identified. We assessed the presence of each reaction in all genomic metabolic networks we reconstructed. By removing reactions absent in any of our genomes, we obtained a set of 3,141 reactions. The presence or absence of each reaction was used as a descriptor for each genome in the form of a fixed length binary vector.

Real cross-feeding and competition interactions were identified through a literature review and database searches based on laboratory-validated interactions. The consortia of two microorganisms were numerically described as two consecutive fixed-length vectors, each representing the metabolism of one of the two interacting microorganisms. To mitigate bias caused by the order of the microbes, we included each interaction in our data set twice, changing the position of each vector from first to second and vice versa. This approach also doubled the number of examples in our data set, resulting in a total of 2,652 pairs.

Furthermore, this data set with 2,652 pairs was divided into four folds of roughly the same size to perform a cross-validation procedure with several machine learning classifiers. These folds, described in Table 1, were created by employing K-means clustering with 10 clusters and default parameters to all 2,652 vectors, made of 6,282 binary elements describing interacting microbes. These 10 clusters were subsequently manually combined to form the four folds employed to train the classifiers. When generating the folds, we ensured that the AB/BA vectors were consistently placed in the same fold to prevent potential data leakage.

### Machine learning methods

Considering the size of our samples, we used a supervised KNN (52–54) algorithm as a baseline to identify cross-feeding and competition interactions between pairs of microorganisms. We set the value of *k* to 3 based on the accuracy (percentage of correctly classified samples) using 30% of one of the folds, with the remaining 70% used for training. This baseline was compared with a Random Forest Classifier (55⁠), four different SVMs (56, 57⁠), and XGBoost (58), all algorithms implemented in Scikit-learn (42).⁠ Quantitative performance comparisons were carried out using precision (P), recall (R), and F1 metrics (59) calculated in the fourfold cross-validation previously mentioned. We also employed receiver operating characteristic and precision recall curves, reporting the area under the curve for each class. To examine the potential compositional effect of our data set, we considered cross-feeding and competition as target classes with all reported metrics.

### Test in Freilich et al. data set

We downloaded the bacterial genomes from the Freilich et al. (7)⁠ data set (composed of 117 different strains, with one of 118 strains described in the study is not available in the public database) from NCBI (Table S2). We then generated genome-based metabolic models for each bacterial genome, following the procedure described in Materials and Methods. Subsequently, we used our predictor (XGBoost model) on the data set to predict the interactions in pairs of bacteria.

We used PCPS between species combinations from Freilich et al. (7⁠). PCPS represents the probability of cross-feeding occurring between pairs of bacteria, with a value of 0 indicating no cross-feeding and a value of 1 signifying cross-feeding. We transformed the matrix, replacing scores over 0 with cross-feeding, scores equal to 0 with no interaction, and negative scores with competition. We compared the matrix using a simple Python script and created a consensus matrix, where the position is +1 if the prediction interaction is cross-feeding in both results, −1 if the prediction is competition consensus, and 0 if there is no consensus.

## Data Availability

All the codes employed in this work are available in https://github.com/networkbiolab/microbia_interaction/ either as standalone scripts or Jupyter notebooks for easy replication of all reported results. The data set and other data files are also available in the same repository.

## SUPPLEMENTAL MATERIAL

Table S1 spectrum.02287-23-s0001.xls List of bacterial genome information used to create the predictor.

Table S2 spectrum.02287-23-s0002.xls Bacterial genome used from Freilich dataset: list of bacterial genome information used to test the predictor.

Table S3 spectrum.02287-23-s0003.xls Interaction information within the primary network cluster.

ASM does not own the copyrights to Supplemental Material that may be linked to, or accessed through, an article. The authors have granted ASM a non-exclusive, world-wide license to publish the Supplemental Material files. Please contact the corresponding author directly for reuse.
