# Correction for Silva-Andrade et al., “Using metabolic networks to predict cross-feeding and competition interactions between microorganisms”

**DOI:** 10.1128/spectrum.01899-24

**Published:** 2024-09-09

**Authors:** Claudia Silva-Andrade, María Rodriguez-Fernández, Daniel Garrido, Alberto J. M. Martin

## AUTHOR CORRECTION

Volume 12, no. 5, e02287-23, 2024, https://doi.org/10.1128/spectrum.02287-23. Page 13, Acknowledgments: “This work was supported by Vicerrectoría de Investigación y Doctorados de la Universidad San Sebastián – Fondo USS-FIN-24-APCS-04” should appear at the end of the second paragraph.

